# ADHD and ASD: distinct brain patterns of inhibition-related activation?

**DOI:** 10.1038/s41398-020-0707-z

**Published:** 2020-01-22

**Authors:** Ariadna Albajara Sáenz, Mathilde Septier, Peter Van Schuerbeek, Simon Baijot, Nicolas Deconinck, Pierre Defresne, Véronique Delvenne, Gianfranco Passeri, Hubert Raeymaekers, Leila Salvesen, Laurent Victoor, Thomas Villemonteix, Eric Willaye, Philippe Peigneux, Isabelle Massat

**Affiliations:** 1 Neuropsychology and Functional Neuroimaging Research Group (UR2NF) at the Centre for Research in Cognition and Neurosciences (CRCN), Université Libre de Bruxelles (ULB), Brussels, Belgium; 2 Hôpital Universitaire Robert Debré, Paris, France; 3 Institut de Psychiatrie et de Neurosciences de Paris Inserm U894 Team 1, Paris, France; 4 Department of Radiology, Universitair Ziekenhuis Brussel (UZ Brussel), Brussels, Belgium; 5 Hôpital Universitaire des Enfants Reine Fabiola (HUDERF), Brussels, Belgium; 6 Service Universitaire Spécialisé pour personnes avec Autisme (SUSA)-Université de Mons, Mons, Belgium; 7 PsyPluriel, Centre Européen de Psychologie Médicale, Brussels, Belgium; 8 Paris 8 Vincennes - St Denis University, Laboratoire de Psychopathologie et Neuropsychologie, Saint Denis, France; 9 Laboratory of Experimental Neurology, ULB, Brussels, Belgium; 10 National Fund of Scientific Research (FNRS), Brussels, Belgium; 11 Department of Neurology, Erasme Hospital, Brussels, Belgium

**Keywords:** ADHD, Autism spectrum disorders

## Abstract

Attention-deficit/hyperactivity (ADHD) and autism spectrum (ASD) disorders often co-occur. In both cases, response inhibition deficits and inhibition-related atypical brain activation have been reported, although less consistently in ASD. Research exploring the overlap/distinctiveness between ADHD and ASD has significantly increased in recent years, but direct comparison of the inhibition-related neuronal correlates between these disorders are scarce in the literature. This study aimed at disentangling the shared and specific inhibitory brain dysfunctions in ASD and ADHD. Using functional magnetic resonance imaging (fMRI), brain activity was compared between children with ADHD, ASD and typically developing (TD) children aged 8–12 years during an inhibition stop-signal task, using stringent inclusion criteria. At the behavioural level, only children with ADHD exhibited inhibition deficits when compared with the TD group. Distinct patterns of brain activity were observed during successful inhibition. In children with ADHD, motor inhibition was associated with right inferior parietal activation, whereas right frontal regions were activated in children with ASD. Between-group comparisons disclosed higher middle frontal activation in the ASD group compared with the ADHD and the TD groups. Our results evidence different patterns of activation during inhibition in these two disorders, recruiting different regions of the fronto-parietal network associated to inhibition. Besides brain activity differences, behavioural inhibition deficits found only in children with ADHD further suggest that reactive inhibition is one of the core deficits in ADHD, but not in ASD. Our findings provide further evidence contributing to disentangle the shared and specific inhibitory dysfunctions in ASD and ADHD.

## Introduction

Attention-deficit/hyperactivity disorder (ADHD) and autism spectrum disorder (ASD) have distinct core diagnostic criteria but often co-occur. ADHD is characterized by impairing levels of inattention and/or hyperactivity–impulsivity, and ASD is defined by deficits in social communication and interaction, and the presence of restricted, repetitive behaviours, interests or activities^1^. Since the publication of the DSM-V, both diagnoses should be given when both ASD and ADHD diagnostic criteria are met^1^. ADHD is the most common comorbid psychiatric condition in referred populations of youths with ASD, with studies reporting comorbidity rates as high as 71%^2,3^. ASD traits are also common in children and adolescents with ADHD, with 12.4% having an ASD diagnosis^4^. Consequently, an increasing number of studies are investigating the overlap and distinctiveness between these disorders at the cognitive, clinical and biological level, to determine whether they are sufficiently distinct to be considered separate disorders, or rather represent the extremes of an underlying continuum^5^. Particularly, the Research Domain Criteria initiative calls for further investigation of transdiagnostic phenotypes and deficits, and their neural underpinnings^6^.

At the neuropsychological level, there is evidence for inhibition deficits in both ADHD and ASD^7,8^. Response inhibition is one of the neurocognitive domains most affected in ADHD, in experimental paradigms such as the stop-signal task (SST)^8,9^, which measures the ability to cancel an ongoing motor response^10^. This paradigm is unique in allowing the estimation of the stop process covert latency: the stop-signal reaction time (SSRT), a sensitive individual index of inhibitory ability^10^. Nevertheless, previous studies using the SST in ASD populations have reported mixed findings, some showing longer SSRTs in ASD compared with typically developing (TD) children^11–14^ and others not^15–19^. Importantly, comorbid ADHD seems to partly explain inhibitory deficits in individuals with ASD^14^.

It is still unclear whether these deficits are driven by the same underlying brain mechanisms. A task-specific meta-analysis evidenced reduced activation in bilateral inferior frontal gyri, the right superior frontal gyrus and the right middle frontal gyrus (MFG) in children with ADHD compared with TD children during the SST^20^. Nevertheless, a recent study evidenced higher activation in a group of non-comorbid medication-naive children with ADHD relative to TD children in regions comprising the fronto-basal ganglio-thalamo-cortical system associated with inhibition^21^.

In ASD samples, only two functional magnetic resonance imaging (fMRI) studies have investigated inhibition-related brain activity using the SST^16,17^. Gooskens et al.^17^ found no differences in activation during successful stopping between children with ASD and TD participants. Instead, higher ADHD symptomatology was associated with increased inhibition-related activation in the left and right frontal and middle cingulate regions, linking changes in cognitive control in ASD to the presence of ADHD symptoms. In a pharmacological fMRI study, Chantiluke et al.^16^ compared inhibition-related brain activation during the SST between non-comorbid ASD, non-comorbid ADHD and TD samples of children and adolescents. In the placebo condition, participants with ASD showed increased activation compared with ADHD in the left middle and inferior frontal cortex (IFC), the left orbitofrontal cortex (OFC), the left superior temporal lobe and the basal ganglia. In addition, opposite disorder-specific brain activation patterns were revealed: there was reduced activation in the left OFC and basal ganglia in the ADHD group, but enhanced activation in the left and right IFC in children with ASD.

It is important to note that fMRI studies using the SST in populations with ADHD and/or ASD vary in their sample characteristics regarding age range, gender, medication history, clinical subtype and/or comorbidities, hindering the characterization of inhibition-related activation patterns in each disorder. In addition, studies differ in their SST design, the within-subject level contrasts, but also the exclusion criteria used based on task performance, hence potentially compromising the validity of the reported results.

To sum up, a growing functional neuroimaging literature has evidenced brain inhibitory dysfunction in both ASD and ADHD. Nevertheless, with only one pharmacological fMRI study directly comparing ASD and ADHD samples^16^, it is deemed too premature to draw conclusions regarding the shared and disorder-specific brain inhibition-related dysfunctions between these disorders. In the present fMRI study, brain activation during the execution of an SST was explored in a sample of children with ASD or ADHD and in a group of TD children. To capture inhibition ability as accurately as possible, SST performance exclusion criteria and analysis were based on recently provided consensus recommendations by Verbruggen et al.^22^ on the correct implementation of the task and data analysis.

Based on previous literature, we hypothesized that (1) children with ADHD and ASD would show longer SSRTs compared with TD children, and that children with ADHD would further exhibit longer SSRTs compared with the ASD sample; (2) children with ASD would show higher activation in the left middle/IFC and right IFC, and children with ADHD would show underactivation in a fronto-basal ganglia network compared with TD children.

## Materials and methods

### Participants

Seventy-four right-handed children aged 8–12 years were enroled in this study. Twenty-nine participants were excluded: 1 participant after the fortuitous discovery of a brain anomaly, 2 participants because of feeling anxious or claustrophobic in the scanner, 18 participants due to inappropriate SST performance and 8 participants for excessive head motion (see below). The final sample consisted of 18 children with ADHD (combined-type), 13 children with ASD and 14 TD children (Table 1). Patients were recruited at the Erasme Hospital Department of Neurology, PsyPluriel-Pastur (European Centre of Psychological Medicine) and autism reference centres at the Queen Fabiola Children’s University Hospital and at the SUSA Foundation (Belgium). TD children had no diagnoses of any psychiatric conditions, did not meet the DSM-V criteria for ADHD or ASD and participated upon announcement or personal query. ADHD and ASD diagnoses were established according to the DSM-V criteria by trained child psychiatrists. ADHD was assessed using the full Kiddie–Sads–Present and Lifetime Version^23^ criteria for ADHD and the parents of all participants responded to the ADHD Rating Scale-IV^24^, assessing the severity of ADHD symptoms. ASD was assessed according to the Autism Diagnostic Interview-Revised, the Autism Diagnostic Observation Schedule and/or the Childhood Autism Rating Scale^25–27^. Exclusion criteria for all participants were as follows: history of prematurity, neurological disorders, genetic disease, complications during labour with neonatal care unit hospitalization, disabling somatic pathology with a potential psychological impact and contraindications to MRI. All participants had a General Ability Index higher than 70 on the Wechsler Intelligence Scale for Children^28^ and one participant with ASD had a Leiter performance score of 74^29,30^. All participants with ADHD were medication-naive and did not present any psychiatric comorbidity. In the ASD group, nine participants also had an ADHD-like comorbidity. In addition, only one participant had a history of psychostimulant (methylphenidate) and antipsychotic (risperidone) intake, and another participant was taking antipsychotic medication at the time of the scanning (Abilify).

**Table 1:** Demographic data and task performance of participants included in the analysis.

	ADHD (*n* = 18)		ASD (*n* = 13)		TD (*n* = 14)		Between-group difference			Post hoc
Gender	M/F 12/6		M/F 9/4		M/F 9/5		*χ*^2^ 0.074	*df* 2	*p* 0.96	NS
	M	SD	M	SD	M	SD	*H*	*df*	*p*	
Age (months)	124.22	18.48	125.62	11.74	133.43	17.05	2.86	2	0.24	NS
IQ	102.56	15.32	107.58	19.94	121.5	16.03	9.03	2	0.01	ADHD < TDC
ADHD RS-IV Total Score	34.44	8.84	21.54	8.65	8.21	6.64	29.73	2	<0.001	ADHD > ASD > TDC
ADHD RS-IV inattention	19.11	4.39	13.38	6.91	5.07	3.67	25.36	2	<0.001	ADHD, ASD > TDC
ADHD RS-IV hyperactivity	15.33	7.00	8.15	4.24	3.14	3.74	22.14	2	<0.001	ADHD > TDC
Head motion CV translation	0.72	0.25	0.66	0.18	0.64	0.25	1.16	2	0.56	NS
Head motion CV rotation	0.72	0.39	0.68	0.25	0.62	0.25	0.41	2	0.81	NS
SSRT (ms)	358.79	115.98	296.23	60.87	273.36	43.90	7.01	2	0.03	ADHD > TDC
Successful stop trials (%)	47.15	6.06	45.58	10.86	43.04	6.59	2.64	2	0.27	NS
MRT (ms)	607.39	129.92	521.78	146.25	472.98	64.8	11.82	2	0.003	ADHD > TDC
SSD (ms)	219.1	86.05	209.23	141.88	167.5	43.36	2.84	2	0.24	NS
Omissions Go trials (%)	9.11	5.86	6.82	2.86	6.51	5.00	2.24	2	0.33	NS
Choice errors Go trials (%)	5.8	4.61	7.11	5.25	4.81	3.6	0.81	2	0.67	NS
RT unsuccessful stop trials (ms)	513.84	100.23	462.29	114.05	417.16	52.01	11.25	2	0.004	ADHD > TDC

*ADHD RS-IV* ADHD Rating Scale-IV, *CV* coefficient of variation, *df* degrees of freedom, *H* test statistic for the Kruskal–Wallis test, *M* mean, *M/F* male/female, *MRT* mean reaction time on successful go trials, *RT* reaction time, *SSD* stop-signal delay, *SSRT* stop-signal reaction time

All participants and their parents gave signed informed consent to participate in this study approved by the Ethics Committee of the ULB-Erasme University Hospital, Brussels, Belgium, and received 50 euros to cover transportation/parking expenses.

### Functional MRI paradigm: the stop-signal task

**Fig. 1:**
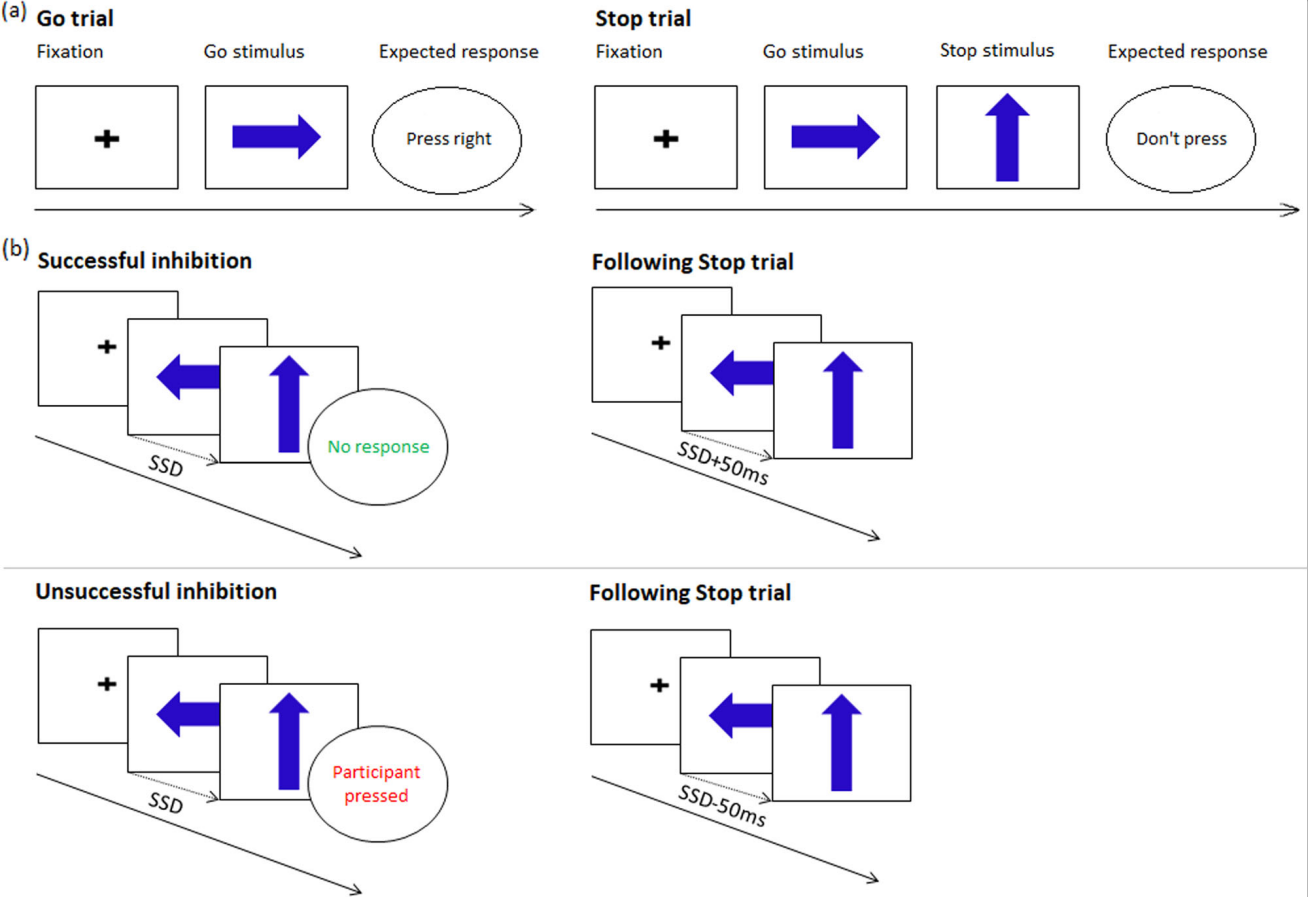
Schematic illustration of the stop-signal task. **a** Participants are instructed to respond as accurately and quickly as possible to go signals, i.e., arrows pointing to the left (*n* *=* 100) or to the right (*n* *=* 100), with a left or right button press according to the direction of the arrow. Display duration depended on participant’s response speed, lasting a maximum of 500 ms. In 25% of the trials, pseudo-randomly interspersed, a stop stimulus (arrow pointing upwards) was presented shortly after the go signal (20 after a right-oriented go stimulus, 20 after a left-oriented go stimulus) and participants were instructed to answer as fast and accurately as possible to the go signal, and to attempt cancelling their ongoing motor response (i.e., not responding) when the stop signal appeared. They were also encouraged not to wait for the stop signal to appear before responding. All trials included a 1000 ms fixed response interval, followed by a variable interval randomly set between 600 ms and 900 ms. **b** Initially, the stop stimulus was displayed 250 ms (Stop Signal Delay, SSD) after the go signal. A tracking algorithm adjusted subsequent SSDs trial-by-trial by steps of 50 ms according to the participant’s performance^22^. If inhibition was successful, then the SSD was made longer by 50 ms to make inhibition of the ongoing response more difficult. If inhibition failed, then the SSD was made shorter by 50 ms to facilitate inhibition. The task involved a total of 200 trials (160 go and 40 stop), requiring a total scan time of ~8 min.

The SST measures the ability to cancel a previously triggered motor response to a go signal, when it is unpredictably followed by a stop signal shortly after. The interval between the go and the stop signals or SSD (Stop Signal Delay) was initially 250 ms, then adapted by 50 ms steps using a horse-race model (i.e., increased when inhibition was successful, decreased when it failed) aimed at an equal distribution of failed and successful inhibition trials (see Fig. 1 for details). SSRT was computed using the integration method^22^, with SSRT = *n*th reaction time (RT) on go trials minus mean SSD, where *n* equals the number of RTs in the RT distribution on go trials multiplied by the overall *p*(respond|signal). Longer SSRTs indicate poorer response inhibition. Other measures of interest for task performance are reported in Table 1. Based on recent consensus recommendations on SST analysis^22,31^, participants meeting any of the following criteria were excluded: percentage of successful stop trials lower than 25% or higher than 75%, percentage of correct responses on go trials inferior to 60% and an RT on unsuccessful stop trials numerically longer than RT on go trials.

### Demographic and behavioural data analysis

Demographic and behavioural data analyses were performed using IBM SPSS Statistics for Windows, version 23 (IBM Corp., Armonk, NY, USA). As part of the demographic parameters and SST behavioural outcome measures were not normally distributed or the assumption of homogeneity of variances was violated, and our sample was relatively small, non-parametric analyses were reported.

### fMRI data acquisition

Imaging data were collected using a Discovery MR750w 3.0T scanner (GE Medical Systems, Milwaukee, WI, USA) at the UZ Brussel hospital (Belgium). A T2*-weighted spin echo planar imaging sequence was used: repetition time (TR) = 3000 ms, echo time (TE) = 70 ms, flip angle = 90°, matrix size = 128 × 128 × 27 and voxel size = 1.88 × 1.88 × 5 mm^3^. Two initial dummy scans were acquired and then discarded for the analysis. Twenty-seven slices were acquired in an ascending and interleaved order, covering the whole brain. Anatomical images were obtained using a T1-weigthed sagittal three-dimensional TFE (turbo field echo) sequence: TR = 8.644 ms, TE = 3.244 ms, inversion time = 450 ms, flip angle = 12°, field of view = 240 × 240 mm^2^, matrix size = 256 × 256 × 128 and voxel size = 0.94 × 0.94 × 1.2 mm^3^. Stimuli were displayed on an MR-compatible backward projection screen visible to the participant through a mirror in the MR head coil. Stimuli were presented using MATLAB (The MathWorks). Motor responses were recorded using a standard MRI-compatible response device (fORP, Current Designs, USA).

### fMRI data analysis

fMRI data were pre-processed and analysed using SPM12 (Statistical Parametric Mapping^32^) implemented in MATLAB2019a (The MathWorks). Pre-processing steps consisted on slice timing correction, realignment, normalization and smoothing with a Gaussian kernel 8 mm full-width at half maximum. Scan-to-scan motion was assessed using ArtRepair^33^. Scans with more than 1.0 mm scan-to-scan movement or more than 1.5% deviation from the average global signal were replaced using a linear interpolation of the neighbouring scan values. Images with total movement > 3 mm were repaired. Participants with more than 30% corrected scans were excluded from further analyses.

For each subject, a first-level intra-individual analysis aimed at modelling data to partition observed neurophysiological responses into components of interest, confounds and error using a general linear model (GLM^32^). To isolate the withdrawal component of inhibition^34^, a contrast map depicting areas of greater activity on “Successful Stop versus Go” trials was created for each subject. Time and dispersion derivatives were included in the model. Twenty-four nuisance motion regressors were added to the GLMs to further control for movement-related effects in fMRI time series^35^. Events were modelled at the time of the go stimulus onset with a duration of 1.5 s^36^. Initial inspection of individual SPM maps revealed no signs of violation of design orthogonality; subsequent calculation indicated that the variance inflation factor was 8.44 for successful stop signal items and 12.48 for successful go items. Individual subject contrasts were entered into a second-level analysis to estimate between-group differences using a one-way analysis of variance (ANOVA). The initial voxel threshold was set to 0.001 uncorrected. For the whole-brain analysis, only clusters with *p*^FWE^-corrected < 0.05 are reported, accounting for multiple comparisons. In addition, a region-of-interest (ROI) analysis was performed in a priori determined locations corresponding to regions reported to exhibit abnormal inhibition-related activation in both ASD and ADHD compared with TD participants, or between-disorders differences, i.e., the bilateral IFC, left inferior parietal lobe, left superior temporal lobe, the right anterior cingulate cortex (ACC), the right posterior cingulate gyrus, the left MFG, bilateral insula, thalamus, caudate and precuneus^16,17,20,21^. ROI masks were created using the Anatomical Automatic Labelling toolbox^37^. Within-mask area inferences were computed using a statistical threshold of *p*^SVC-FWE^ < 0.05 at the peak level. The *p*-value was Bonferroni-adjusted for the number of regions examined: *p*^SVC-FWE^ = 0.05/10 = 0.005. Extraction of β-values and percentage signal change for illustrative purposes was made using RFXplot^38^. SSRT values (Table 1) were entered as a covariate in the analyses. As lower IQ is a feature in ADHD and ASD making statistical control impossible, and does not meet the requirements for a covariate^39^, all analyses were conducted without IQ as a covariate.

## Results

### Demographic data and head motion

Gender was not significantly related to diagnosis and groups did not differ on age (Table 1). IQ was significantly higher in the TD group compared with the ADHD (*p* = 0.01), which is typical in this population^9^. Groups differed significantly on the ADHD-RS-IV scores (Total, Inattention and Hyperactivity–Impulsivity). The ADHD group total score was significantly higher compared with that of ASD (*p* = 0.018) and TD children (*p* < 0.001) scores. The total score was also significantly higher for the ASD group compared with that of the TD group (*p* = 0.045), due to the presence of ADHD comorbidity in nine participants (Supplementary Table 1). The inattention score in the TD group was significantly lower compared with that of both the ADHD (*p* < 0.001) and the ASD groups (*p* = 0.016). The hyperactivity score was significantly higher in the ADHD group compared with that of the TD group (*p* < 0.001). Finally, there were no significant differences between groups regarding head motion. Additional analyses including non-comorbid ASD and ASD with ADHD comorbidity separately are reported in the Supplementary Materials.

### Stop-signal task performance

Contrary to children with ASD, the ADHD group showed significantly longer SSTRs (*p* = 0.029), MRTs (*p* = 0.003) and mean RT on unsuccessful stop trials (*p* = 0.004), as compared with the TD group. No other significant differences were found for task performance (Table 1).

### Within-group brain activations during successful inhibition

**Fig. 2:**
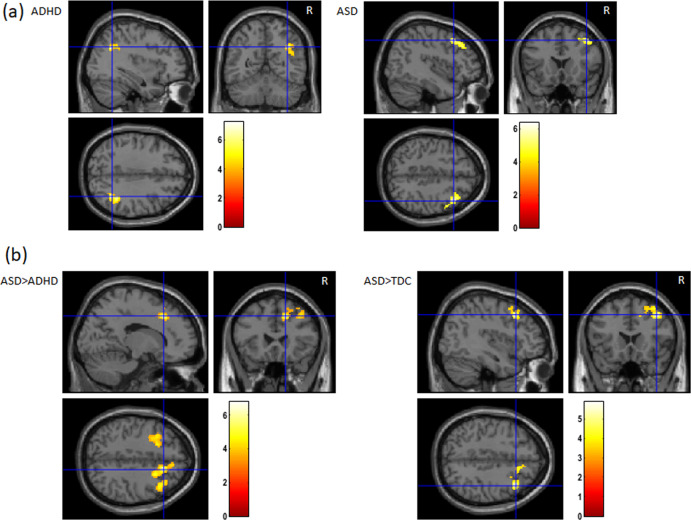
Brain activation during Successful Stop versus Go at the cluster level *p*^FWE^ < 0.05. **a** Within-group activation in a cluster comprising the right angular gyrus/intraparietal sulcus and inferior parietal gyrus in the ADHD group (*x* = 36, *y* = −56, *z* = 34; *p* < 0.001) and in the right middle frontal gyrus in the ASD group (*x* = 42, *y* = 18, *z* = 46; *p* < 0.001). **b** Between-group comparison showing higher activation in the right cingulate gyrus/middle frontal gyrus and the left middle frontal gyrus in children with ASD compared with the ADHD group and in the right middle frontal gyrus in children with ASD compared with the TD group.

The whole-brain analysis of the Successful Stop versus Go contrast disclosed increased activity (*p*^FWE^ < 0.05) in a cluster comprising the right angular gyrus, the right inferior parietal cortex and the intraparietal sulcus in the ADHD group (Table 2 and Fig. 2a). In the ASD group, the same contrast revealed activation in the right MFG (*p*^FWE^ < 0.05). For these whole-brain analyses, results were comparable with or without age entered as a covariate. The TD group did not show activation at *p*^FWE^ < 0.05 (whole-brain analysis).

**Table 2:** Within-group activation and between-group comparison of activation during successful inhibition.

Contrast	Hemisphere	Anatomical region	MNI coordinates peak voxel			Cluster size *k*	Cluster *p*-value
			*x*	*y*	*z*		
ADHD	R	Angular gyrus, inferior parietal lobe, intraparietal sulcus	36	−56	34	371	<0.001^a^
	R	Posterior cingulate gyrus	6	−34	22	13	0.001^b^
ASD	R	Middle frontal gyrus	42	18	46	411	<0.001^a^
ASD > ADHD	R	Cingulate gyrus, middle frontal gyrus	16	24	42	852	<0.001^a^
	L	Middle frontal gyrus	−22	16	42	232	0.018^a^
ASD > TD	R	Middle frontal gyrus	40	20	44	564	0.020^a^
ADHD > TD	R	Posterior cingulate	4	−42	20	18	0.003^b^

^a^Significant activation clusters at FWE-corrected *p* < 0.05 (whole-brain analysis). ^b^Significant activation in ROI after Bonferroni correction at *p*^SVC-FWE^ < 0.005 at the peak level (ROI analysis). *L* left hemisphere, *R* right hemisphere.

The ROI analysis revealed a significant effect after Bonferroni correction in the right posterior cingulate cortex in the ADHD group (*p*^SVC-FEW^ < 0.005). No ROI survived Bonferroni correction within the ASD and the TD group.

### Between-groups comparison

**Fig. 3:**
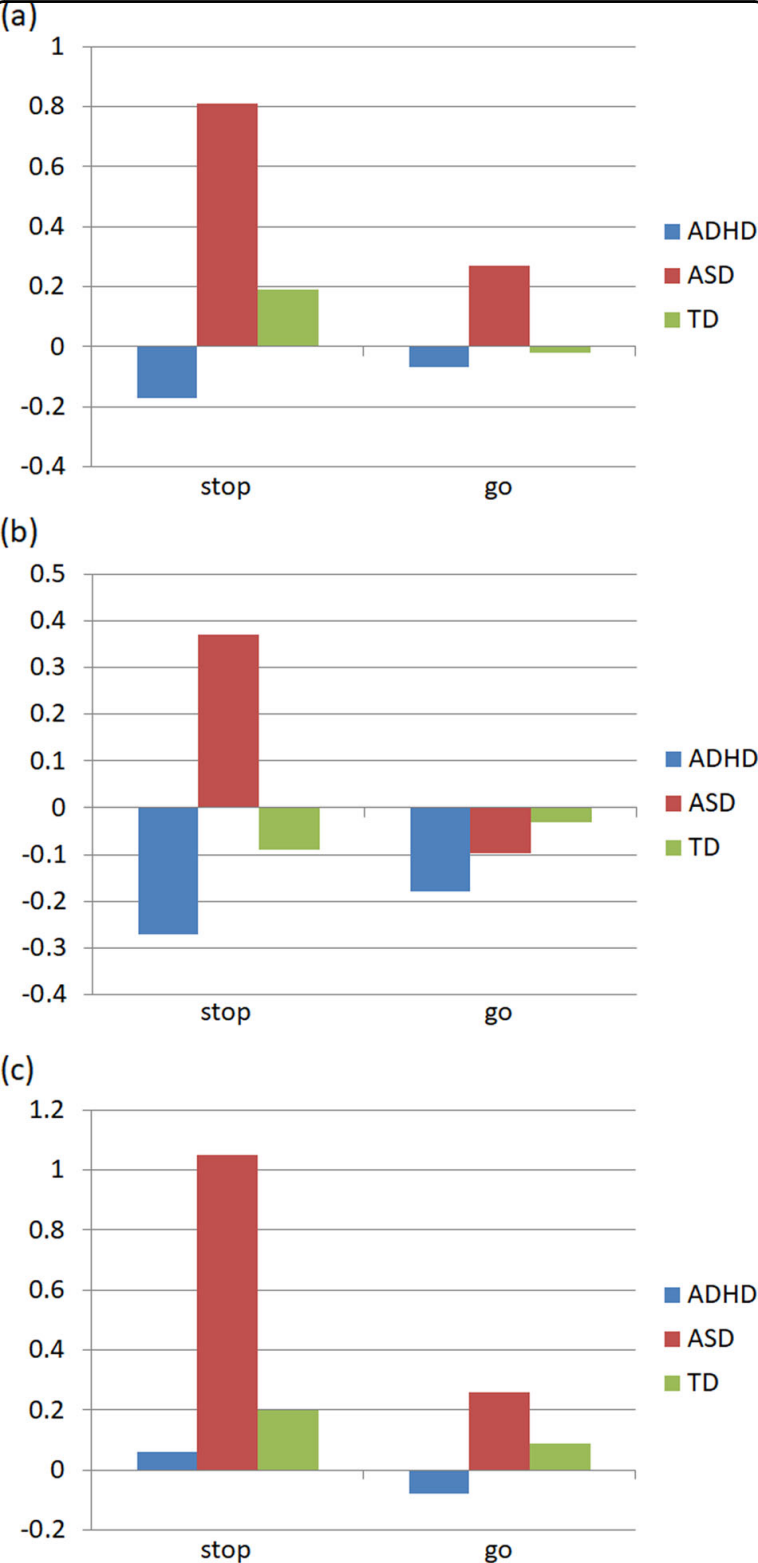
Percent signal change in activation during successful stop and go in regions showing between-group significant differences: **a** the right cingulate gyrus/ right middle frontal gyrus (*x* = 16, *y* = 24, *z* = 42), **b** the left middle frontal gyrus (*x* = −22, *y* = 16, *z* = 42) and **c** the right middle frontal gyrus (*x* = 40, *y* = 20, *z* = 44).

*Whole-brain analyses*: A one-way ANOVA analysis (Table 2 and Fig. 2b) revealed a significant group effect in the right cingulate/MFG at the cluster level *p*^FWE^ < 0.05 (*p* = 0.030; cluster size = 152) and peak-level *p*^FWE^ < 0.05 (*p* = 0.017; *x* = 16, *y* = 24, *z* = 42 mm). Pairwise comparisons showed that the group effect in this region was due to a significant higher activation in the ASD group during successful inhibition compared with the ADHD group (*p* = 0.002, peak-level *p*^FWE^ < 0.05; Fig. 3a). Higher activation in the ASD group during successful inhibition compared with the ADHD group was also found in the left MFG at the cluster level *p*^FWE^ < 0.05 (*p* = 0.018; *x* = −22; *y* = 16; *z* = 42; Fig. 3b). Finally, we found higher activation in the right MFG in the ASD group compared with the TD participants at the cluster (*p*^FWE^ < 0.001; cluster size = 564) and peak level (*p*^FWE^ = 0.020; *x* = 40, *y* = 20, *z* = 44 mm) during successful inhibition (Fig. 3c). Results for these analyses were comparable with or without age entered as a covariate. In an exploratory analysis, a one-way ANOVA with four groups was subsequently performed, in which the ASD sample was divided into two groups: non-comorbid ASD (*n* = 4) and ASD with ADHD comorbidity (*n* = 9). A small volume correction with a 10 mm sphere radius centred on the three coordinates reported above was examined at the peak-level *p*^SVC-FWE^ < 0.05. Activations remained significant in all these regions, suggesting that between-group differences were not explained by the presence of comorbid ADHD in nine patients with ASD.

Finally, conjunction analysis did not show common activations between the patients groups.

*ROI analyses*: Analyses conducted on ROIs disclosed higher activation in the ADHD than the TD group in the right posterior cingulate gyrus (*p*^SVC-FWE^ < 0.005; *x* = 4, *y* = −42, *z* = 20 mm) after Bonferroni correction. No other ROIs survived Bonferroni correction.

## Discussion

In this study, both inhibition deficits and slower responding were found in the ADHD as compared with the TD group at the behavioural level, consistent with previous studies^8,9^. No differences in task performance were found between children with ASD and TD children, consistent with Schmitt et al.^19^, who found that the ability to reactively stop behaviours as measured by the SSRT was preserved in a group of children and adults with ASD. Nevertheless, in their study, RT slowing during go trials was reduced in patients compared with controls, suggesting a deficit in strategically delaying the onset of behavioural responses, i.e., proactive control.

fMRI analyses revealed different activation patterns in children with ASD and ADHD when explored separately, even if nine participants in the ASD group had an ADHD-like comorbidity. Conjunction analysis did not show common activations between the patients groups, emphasizing the between-group distinctiveness in activation patterns. In the ADHD group, successful inhibition activated a cluster comprising the right angular gyrus, the intraparietal sulcus and the inferior parietal gyrus, in line with previous studies in children and adolescents with ADHD^40,41^. These regions have been associated with action cancellation and action withholding^42^.

In children with ASD, activation was found mostly in frontal regions, in particular, the right MFG, also associated with action withholding and cancellation^42^. These results show that response inhibition may place different demands on the fronto-parietal network in ASD and ADHD, eventually activating regions that serve different functions in response inhibition. Although the inferior parietal cortex has been associated with rapid movement prevention^43^ and attentional capture of infrequent stimuli^44^, its contribution to inhibition per se remains unclear. The recruitment of parietal nodes in children with ADHD suggests they may need a higher involvement of attentional processes for successful inhibition than children with ASD, thereby modulating the response inhibition process.

Between-groups comparisons disclosed higher activation in the ASD than the ADHD group in the right cingulate gyrus/MFG and the left MFG. These results are consistent with Chantiluke et al.^16^, who also found increased activation in the left middle/IFC in boys with ASD as compared with those with ADHD, for a comparable task performance. In addition, there was also higher activation in the right MFG in the ASD group as compared with the TD group during successful inhibition. These results suggest that children with ASD may require recruiting more of these brain areas to achieve a task performance comparable to the ADHD and the TD groups. Differences observed in the left frontal cortex indicate that the left hemisphere should not be neglected in the study of response inhibition in ASD, as previously suggested^45^.

Regarding the ACC, ROI analysis revealed higher inhibition-related activity in the ASD than in the TDC and the ADHD groups, in agreement with previous studies evidencing atypical brain activity during response inhibition in autism^45–47^. However, this difference did not survive Bonferroni correction and the result should thus be considered with caution. The ACC has been associated with conflict and performance monitoring and salience detection during response inhibition^48,49^. Finally, ROI analysis revealed inhibition-related differences between the ADHD and TD groups in posterior cingulate activity, which is consistent with recent preliminary meta-analysis results^50^.

The findings in this study should be considered in the light of some limitations. First, our final sample was relatively small due to strict exclusion criteria regarding head motion and/or non-adequate SST performance. The strict but necessary exclusion task performance criteria were based on the latest consensus recommendations by Verbruggen et al.^22^ to analyse SST data in such a way that the ability to inhibit is accurately captured. Such precautions have often been dismissed or have been applied in a more lenient way in previous studies. Therefore, we argue that the strict exclusion criteria used actually represents the strength of our study, admittedly at the cost of a reduced sample size. A related limitation is that our sample was only composed of individuals able to perform the task inside of the scanner and whose data were included after data quality control regarding task performance and head motion. Consequently, the results of our study may not be generalizable to all individuals with ASD and/or ADHD, and especially those who display more severe ASD and/or ADHD symptoms or exhibit lower cognitive abilities. Our gender balanced sample was however large enough to detect robust significant brain activation differences between groups in our whole brain analysis, even after correction for multiple comparisons. In addition, all children with ADHD were non-comorbid and medication naïve in order to avoid drug-induced brain changes previously evidenced in other studies^16^ and only one participant with ASD had a previous history of psychostimulant intake. Therefore, there was no potential confounding effect of medication and/or comorbidity on inhibition-related activity in the ADHD sample. Again however, strict inclusion criteria may hinder the generalization of our results to the heterogeneous population of ADHD patients who present various comorbidities and/or medication status in clinical practice. Finally, the power of our statistical design was somehow limited by the use of short inter-stimuli intervals including jitters (leading to increased variance inflation factors) aimed at keeping the task duration as short and motivating as possible for our specific populations, whose inclusion in fMRI studies remain a challenge.

Finally, there was a high percentage of children in the ASD group, who had an ADHD comorbidity, which is consistent with recent comorbidity reports^2,3^, but potentially confounding our findings. Nevertheless, similar results were found regardless of ADHD comorbidity. The distinct patterns of activation between disorders suggest that ADHD-like symptoms in ASD are associated with a different pattern of activation compared with non-comorbid ADHD and raise the question of the distinctiveness between ADHD-like symptoms present in ASD and pure ADHD, which constitutes a crucial clinical insight in the study of the comorbidity between ASD and ADHD.

To conclude, a distinct pattern of activation during successful inhibition in a sample of children with ADHD and a sample of children with ASD was evidenced for the first time using the SST, despite the presence of ADHD-like symptoms in nine participants in the ASD group. Children with ADHD showed activation in inferior parietal regions and children with ASD in bilateral middle frontal regions during successful inhibition as compared with go trials. The ASD group showed higher activation in the middle frontal cortex compared with TD participants, whereas the ADHD showed higher activation than the TD group in the right posterior cingulate. Importantly, only the ADHD and not the ASD group showed behavioural inhibition deficits compared with the TD group, reinforcing the idea that reactive inhibition is one of the core deficits in ADHD, but not in ASD. Future work should explore the commonalities and distinctions in inhibition-related activity in a larger sample of children with ASD fractionating the sample in terms of the presence or absence of ADHD-like symptoms and a sample of children with ADHD, to examine the differences between the ADHD-like comorbidity in ASD and a primary diagnosis of ADHD.

## Supplementary information

Supplementary Material
