# Supplementary material for: ADHD and ASD: distinct brain patterns of inhibition-related activation?
Source: Transl Psychiatry. 2020 Jan 22;10:24. doi: 10.1038/s41398-020-0707-z (PMC7026183; doi:10.1038/s41398-020-0707-z)
Supplement: Supplementary file 1 — Supplementary Material [file 41398_2020_707_MOESM1_ESM.pdf]

Supplementary Material Table 1. Demographic data of participants included in the analysis.

	ADHD (n=18)		Pure ASD (n=4)		Comorbid (n=9)		TD (n=14)		Between-group difference			Post hoc
	M/F		*M/F*		*M/F*		*M/F*		$\chi2$	*df*	*p*	
Gender	12/6		2/2		7/2		9/5		0.074	2	0.96	ns
	M	*SD*	*M*	*SD*	*M*	*SD*	*M*	*SD*	*H*	*df*	*p*	
Age (months)	124.22	18.48	126.5	7.33	125.22	13.64	133.43	17.05	2.89	3	0.41	ns
IQ	102.56	15.32	95.00	28.58	111.78	16.22	121.50	16.03	9.75	3	0.02	ADHD<TD
ADHD RS-IV Total Score	34.44	8.84	11.25	5.44	26.11	4.96	8.21	6.64	32.81	3	<0.001	ADHD> pure ASD,TD<br>Comorbid>TD
ADHD RS-IV Inattention	19.11	4.39	5.75	2.06	16.78	5.29	5.07	3.67	30.68	3	<0.001	ADHD>pure ASD,TD<br>Comorbid>TD
ADHD RS-IV Hyperactivity	15.33	7.00	5.5	4.43	9.33	3.81	3.14	3.74	23.09	3	<0.001	ADHD>TD

Note. M/F= Male/Female; ADHD RS-IV= ADHD Rating Scale-IV; $\chi2$= Pearson's Chi-squared test; df= degrees of freedom; p= p-value; SD= Standard deviation; H: test statistic for the Kruskal-Wallis test.
